# Improving the Post-Operative Prediction of BCR-Free Survival Time with mRNA Variables and Machine Learning

**DOI:** 10.3390/cancers15041276

**Published:** 2023-02-17

**Authors:** Autumn O’Donnell, Eric Wolsztynski, Michael Cronin, Shirin Moghaddam

**Affiliations:** 1 School of Mathematical Sciences, Western Gateway Building, Western Road, University College Cork, T12 XF62 Cork, Ireland; 2 Insight SFI Centre for Data Analytics, Western Gateway Building, Western Road, University College Cork, T12 XF62 Cork, Ireland; 3 Department of Mathematics and Statistics (MACSI), University of Limerick, V94 T9PX Limerick, Ireland

**Keywords:** machine learning, survival analysis, prediction, statistical modelling, genomics, prostate cancer, biochemical recurrence, personalised medicine, bioinformatics

## Abstract

**Simple Summary:**

Prostate cancer is among the most prevalent cancers for men globally, accounting for 13% of cancer diagnoses in the male population each year. Surgical intervention is the primary treatment option but fails in up to 40% of patients, who experience biochemical recurrence (BCR). Determining the likelihood of recurrence and the length of time between surgery and BCR is critical for patient treatment decision-making. Traditional predictive models exploit routine clinical variables such as cancer stage, and may be improved upon by leveraging other accessible information about the patient. This study considers including patient-specific genomic data to identify relevant additional predictors of BCR-free survival, which requires the use of modern machine learning techniques. The results of this study indicate that including such genomic data leads to a gain in BCR prediction performance over models using clinical variables only.

**Abstract:**

Predicting the risk of, and time to biochemical recurrence (BCR) in prostate cancer patients post-operatively is critical in patient treatment decision pathways following surgical intervention. This study aimed to investigate the predictive potential of mRNA information to improve upon reference nomograms and clinical-only models, using a dataset of 187 patients that includes over 20,000 features. Several machine learning methodologies were implemented for the analysis of censored patient follow-up information with such high-dimensional genomic data. Our findings demonstrated the potential of inclusion of mRNA information for BCR-free survival prediction. A random survival forest pipeline was found to achieve high predictive performance with respect to discrimination, calibration, and net benefit. Two mRNA variables, namely ESM1 and DHAH8, were identified as consistently strong predictors with this dataset.

## 1. Introduction

Radiation prostatectomy (RP) is the primary treatment for prostate cancer [1]. Approximately 30% of patients undergo RP [2], of which up to 70% remain disease-free after 10 years [3], while 20–40% fail and experience biochemical recurrence (BCR) [4]. In the latter case, the time from surgical intervention until BCR varies across patients, with over 60% of BCR cases occurring in the first two years post-operatively [5]. The probability of BCR-free survival is a widely investigated topic as it plays a role in patient treatment decision making [6,7]. The probability of recurrence can be used to determine if and when adjunctive therapies, such as radiotherapy, hormone therapy, and chemotherapy, should be initiated [8]. Current state-of-the-art nomograms such as that produced by the Memorial Sloan Kettering Cancer Centre (MSKCC) use routine clinical variables to make the prediction [9].

Originally developed by Kattan et al. in 1999, a post-operative nomogram using prostate specific antigen (PSA) at diagnosis, Gleason sum score, prostatic capsular invasion, surgical marginal status, seminal vesicle invasion, and lymph node status are used to make prediction of BCR at seven years [10] with updates on this in 2005 and 2009 [11,12]. Other models such as the Walz et al., 2009, are designed for prediction at 2-years post-operatively [5]. Two potential limitations of these reference nomograms are the modelling structure they are based on and the nature of the variables used. Nomograms can predict only for the probability of an event occurring at a specific point in time; the original Kattan nomogram [10], for example, considers a 7-year post-RP horizon. This limits the ability to make treatment decisions at different time-points. Both nomograms also consider only clinical variables, although further information may become routinely available for prediction. This study considered models that allowed for less stringent assumptions on the variable effects, and the inclusion of a number of genetic variables, to overcome these limitations.

The Cox proportional hazard model [13] is a statistical approach of reference for the analysis of censored survival data. Two key assumptions of this model that may be unrealistic are that the independent risk predictors interact linearly in quantifying the relative risk of patients, and that all hazards are proportional within the cohort. Any continuous predictor variable with a non-linear relationship to the risk would need to be transformed to fit the model. However, this would require prior knowledge of the distribution of all variables to be considered for inclusion [14]. Furthermore, such traditional regression models are prone to overfitting, in that the addition of (possibly redundant) variables to the model can lead to an improved fit whilst reducing the generalisability of the model to unseen patients [14,15]. Other important limitations of Cox models in the context of modern, high-dimensional clinical datasets, are their inability to adjust for collinearity (i.e., redundancy) in the feature set, and their non-applicability to datasets containing more candidate predictors than observations [15,16].

Machine learning methodologies offer solutions to overcome these limitations. Model regularisation can be applied to traditional models including Cox models [17] to handle collinearity and reduce the potential of overfitting, yielding the likes of the Cox lasso and Cox ridge regression models [15]. Gradient boosting may also be applied to Cox modelling in order to mitigate the risk of overfitting, and so, is naturally applicable in high-dimensional environments [18,19]. Other alternative models include the random survival forest, a non-parametric, tree-based strategy that does not impose linearity constraints and thus allows the inclusion of covariates with associations that are not linear in nature [20].

Improvements may be achieved also in terms of the nature of the information used in current clinical nomograms, since these use routine clinical variables only. Over the past 15 years, the utility of gene expression biomarkers has been widely researched and demonstrated [21,22,23,24,25]. The assessment of mRNA variables has become more cost-reasonable and feasible, offering new opportunities to improve on current models. Currently, mRNA data typically becomes available post-operatively when clinicians have large biological samples of diseased tissue to extract the genetic component from. The primary goal of this study is, firstly, to determine whether the inclusion of mRNA variables can lead to an improvement in the predictive performance of models determining time-to-BCR, and secondly, to identify relevant statistical or machine learning methodologies to perform this prediction.

## 2. Materials and Methods

### 2.1. Data

The dataset used for this analysis was developed in a study by Taylor et al. and contains a total of 232 patients [25] and is comprised of 2 parts, i.e., a set of routine clinical variables and another of messenger ribonucleic acid (mRNA) variables. Following pre-processing for duplicates and missing information, a final 187 patients with complete information including BCR-free duration were retained. BCR in this dataset, is defined as PSA ≥ 0.2 ng/mL on two occasions post-operatively [25]. Any records of a Gleason score of 5 were upgraded to a score of 6 per the current grading system [26]. Of these 187 remaining patients, 133 had mRNA information, and so any mRNA analysis was carried out on this sub-cohort. Although the primary and secondary Gleason scores were available for all remaining individuals, they were not considered for use in our model pipelines as their combined information is already captured in the combined Gleason sum score. Pre-operative variables such as clinical stage and biopsy Gleason score were also excluded as these are seen as approximations of the pathological variables available for analysis in the post-operative setting. As extracapsular extension (ECE), seminal vesicle invasion (SVI), and lymph node involvement (LNI) were included in the modelling, the pathological stage was also removed as the combined effect of these variables should account for all of its effect [27]. All of the aforementioned variable exclusions are in line with the method of the original Kattan nomogram and the current, state-of-the-art MSKCC nomogram [9,10].

The final set of routine clinical variables consisted of both continuous and categorical features. The continuous variables were PSA (mean = 10.4, standard deviation (SD) = 16.13) and age (mean = 58.1, SD = 6.67) at diagnosis and the categorical variables considered for inclusion in a model can be seen in Table 1 along with their descriptive statistics. The set of mRNA variables was not pre-processed, and was comprised of over 20,000 features.

### 2.2. Statistical Analysis

Four distinct predictive modelling strategies were considered: the traditional Cox proportional hazard (CPH) model [13]; an $L_{1}$-penalised regularisation of this model named LASSO Cox [28]; a gradient boosting approach consisting of a sequential ensemble of CPH models, named Boosted Cox [29]; and a random survival forest (RSF) [20], as an alternative to Cox-based modelling of the risk of BCR. Each modelling approach was implemented with either routine clinical variables only, or with a combination of clinical and mRNA variables.

Before inclusion, the set of mRNA variables was reduced using pre-filtering and feature selection techniques outlined hereafter, applied either separately or combined, prior to model building. A correlation-based pre-filter was considered in order to remove variables with a high Pearson correlation with other variables from the dataset in order to prevent multicollinearity, using an absolute cut-off value of 0.6. This cut-off was selected as it had previously been shown to be optimal by Goh et al. on similar data [30]. The feature selection technique consisted of performing univariate Cox PH analysis on each variable separately and ordering the output with respect to their adjusted log-rank score, whilst also adjusting all *p*-values for false discovery rate (FDR) [31]. The 50 variables with an FDR-adjusted *p*-value less than 5% and yielding highest log-rank scores were retained for inclusion for multivariate modelling. This method was implemented in line with that outlined by Beer et al. on their assessment of gene expression and the prediction of lung cancer [32]. Such multiple testing methods can be prone to high false positive rates [33], and FDR *p*-value correction is a popular method used to mitigate this risk. All traditional CPH models underwent further feature selection by means of a forward stepwise selection with a maximum of 10 variables chosen for the models with mRNA variables. All RSF models underwent recursive feature elimination [34] with the selected number of variables being evaluated based on the model yielding the highest concordance index (C-index), which is defined as the proportion of all usable pairs of patients whose predicted and observed outcomes are concordant [35,36].

R (version 2022.07) [37] was used for all statistical and machine learning analyses. The regularisation parameter (λ) was selected using cross-validation for the LASSO Cox models [38], using a final value for λ corresponding to one standard deviation of the cross-validated error, as a standard approach to achieve parsimony [34]. Hyperparameters for the boosted Cox and RSF models were kept to default settings [29,39]. Thus, boosted Cox models contained 100 boosting iterations and a fixed shrinkage parameter of 0.1. The RSF models were built using 500 trees, considering $\sqrt{P}$ variables at each split (where *P* denotes the number of features considered for modelling) and with a minimum size of 15 for the terminal nodes.

### 2.3. Evaluation Framework

The models were assessed and bench-marked via bootstrapping, using 100 bootstrap resamples for model building and the corresponding out-of-bag (OOB, i.e., test) points for independent testing. Thus, training samples of size N = 133, sampled from the original dataset with replacement, were used. Those observations not used in the training sample were used in the independent test samples, each of size n = 49. The predictive performance of the models was assessed in terms of discrimination and calibration. In this study, discrimination refers to the model’s ability to correctly rank patients relative to each other, using a bootstrap-corrected c-index and corresponding confidence intervals (CI), in order to obtain unbiased estimates of external predictive discrimination [40]. Calibration analysis provides a way to assess model bias for various risk profiles, by comparing the observed and predicted probabilities [41]. Here, two approaches to calibration were implemented within the bootstrapping framework. The method of Harrell et al. compares quintiles of the predicted survival of patients at a specific time point to their Kaplan–Meier estimates [40]. Two- and five-year BCR-free survival endpoints were considered in keeping with calibration of the reference MSKCC nomogram. The second method involves the comparison of quintiles of the predicted overall BCR-free survival probability with their observed Kaplan–Meier estimates [42]. This second method allows for inspection of the model’s ability to predict across all time points, as opposed to the 2-year or 5-year survival horizons.

Receiver operating characteristic (ROC) curve and decision curve analysis (DCA) were also assessed at both 2-year and 5-year horizons to further assess the predictive performance. ROC analysis was summarised in terms of the corresponding bootstrapped area under the ROC curve (AUC) values calculated on the OOB points [36,43]. DCA [44] provided complementary assessment in terms of the clinical value and potential net benefit to patients of the models’ predictions.

In complement to predictive performance, the feature selection stability of the best-performing modelling pipelines across the 100 bootstraps was assessed in terms of the overall feature selection rates, and measured using the Jaccard, Davis, and Dice indices [45,46]. These indices range between 0 and 1, a higher index value indicating a more stable model [47]. Further, individual feature selection rates were analysed to extract information about potentially relevant predictors for BCR.

## 3. Results

The results from the evaluation of the predictive pipelines described in Section 2.2 using the frameworks outlined in Section 2.3 are reported in this section.

### 3.1. Discrimination

**Figure 1:**
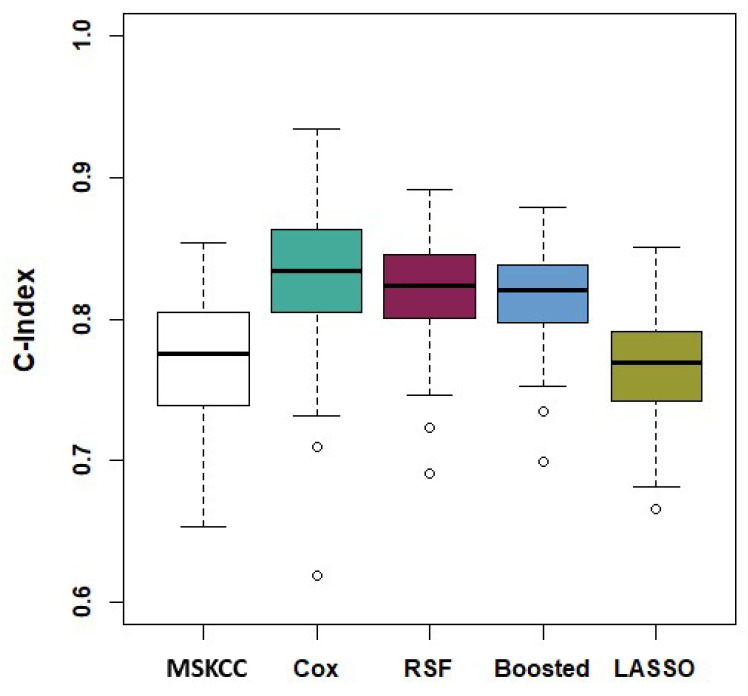
Bootstrap C-Index distributions of the MSKCC nomogram and the most competitive model pipelines using mRNA data. From **left** to **right**; MSKCC, Cox model with clinical variables and correlation prefiltered mRNA variables, RSF model with clinical variables and correlation prefiltered and univariate Cox feature selected mRNA, Boosted Cox model with clinical variables and correlation prefiltered mRNA variables, LASSO Cox model with clinical variables and univariate Cox feature selected mRNA.

The models’ discriminatory performance based on the bootstrapped C-indices are shown in Table 2 along with their corresponding confidence intervals. The boxplots of the performing pipelines are shown in Figure 1.

Pairwise two-sided Wilcoxon signed-rank tests of the null hypothesis of no difference in the C-index distributions reported in Table 2 were carried out on all pairs of the models, in order to compare the discriminative performance of the pipelines relative to each other, with FDR correction for *p*-values [31]. The traditional CPH model and the RSF using only clinical variables outperformed the MSKCC nomogram (*p*-value < 0.001). No significant difference was found between the MSKCC and either the LASSO or Boosted models (*p*-value = 0.210 and 0.915, respectively). There was also no significant difference between the traditional CPH model and the RSF (*p*-value = 0.280). These tests also indicated that at least one of the models that included mRNA variables outperformed each of the clinical-only models. Furthermore, the inclusion of mRNA variables yielded improved performance for the CPH, Boosted Cox, and RSF models (all *p*-values < 0.001), but not for the LASSO Cox model (all *p*-values > 0.300). For all modelling strategies, the pipeline yielding the highest bootstrapped C-index was identified as the best-performing pipeline (shown in bold in Table 2) and retained for further analyses.

### 3.2. Calibration

**Figure 2:**
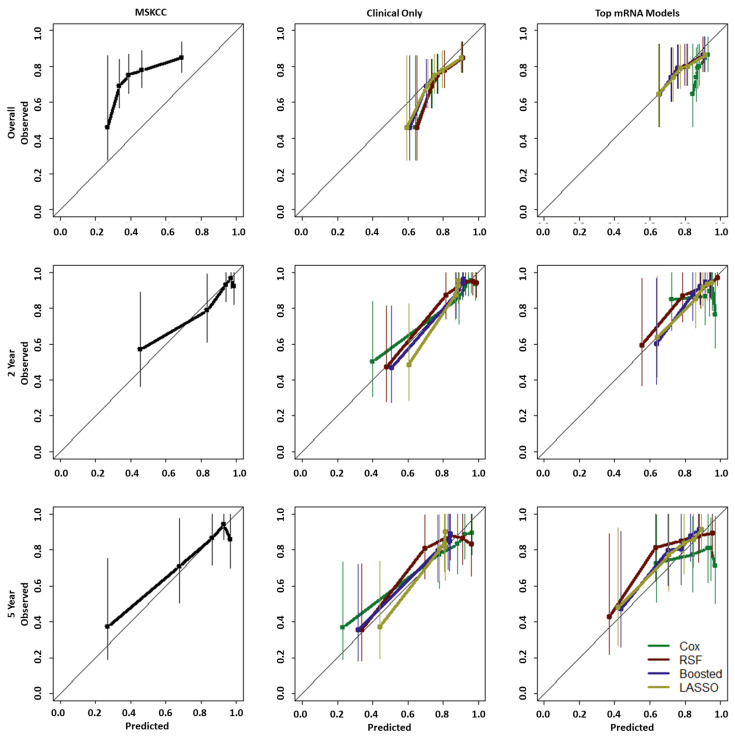
Calibration curves for MSKCC (**left**), clinical-only models (**centre**) and best-performing mRNA-based models (**right**) for (from **top** to **bottom**) overall, 2-year and 5-year survival.

The MSKCC nomogram, the current state-of-the-art post-operative model, is designed with a 2-year or 5-year horizon as the predictive endpoints, and all models were tested for both 2- and 5-year survival calibration, as shown in Figure 2. For this data, the MSKCC nomogram showed reasonable calibration for both these endpoints. Some under-prediction occurred for those with low observed survival and some over-prediction occurred for those with higher observed survival. For overall survival, however, the nomogram appeared mis-calibrated for this data, yielding under-prediction at all time points of reference.

In the clinical-only models, overall survival was slightly over-predicted but with much closer adherence to the 45-degree line overall (thus, closer to the observed values) than the MSKCC model. All clinical-only models also showed reasonable calibration at the 2- and 5-year endpoints. It can be noted that in both 2- and 5-year endpoint curves the LASSO and Boosted Cox models yielded very little separation between the top four quintiles.

As for calibration of the best-performing mRNA models; RSF, Boosted, and LASSO models all yielded similar calibration curves with close adherence to the 45-degree line for overall survival as well as 2- and 5-year survival. The CPH model, however, showed poorer calibration for all three endpoints. Thus, optimisation of the CPH model with respect to the C-index, with the inclusion of mRNA variable, led to a decrease in model calibration.

### 3.3. Prediction Performance

**Figure 3:**
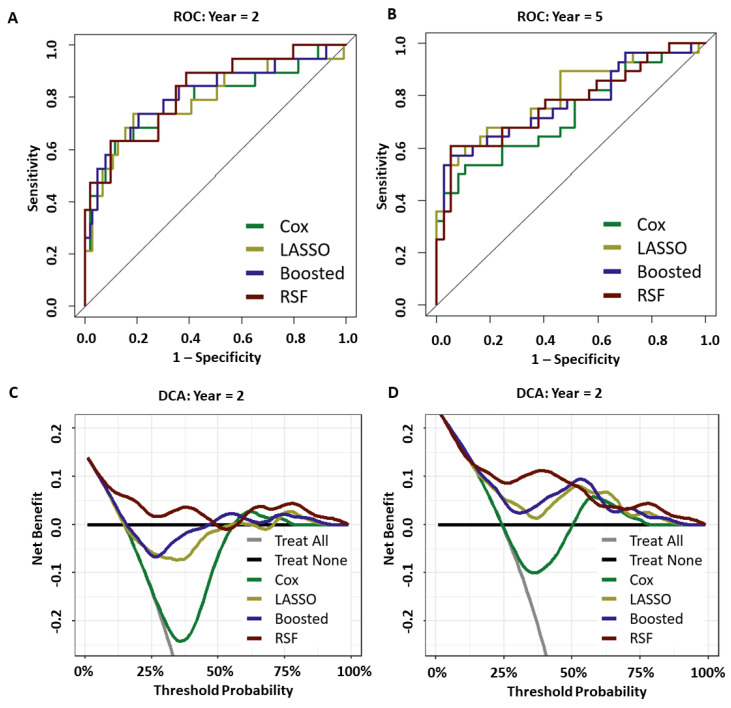
(**A**,**B**): ROC for 2-year (**A**) and 5-year (**B**) horizons. (**C**,**D**): DCA curves at the same horizons.

ROC and decision curve analysis (DCA) were also both carried out for the 2- and 5-year survival endpoints, with the output shown in Figure 3. Optimal out-of-bag prediction [48] was achieved by the RSF at the 2-year horizon (AUC = 0.824, CI of 2000 bootstrap resamples = (0.716, 0.933)) and 5-year horizon (AUC = 0.772, CI = (0.651, 0.893)). DCA indicated that only the RSF model showed a continued net benefit across all threshold probabilities for both the 2- and 5-year endpoints. All models improved upon the “Treat All” method, but none of the other methodologies showed benefit at 2 years. At 5 years both the LASSO and Boosted Cox models retained some net benefit across all thresholds but were below that of the RSF model.

### 3.4. Model Stability and Feature Selection

Overall model stability was evaluated on all clinical-only pipelines and on the best performing pipelines for each modelling strategy when including the entire set of over 20,000 mRNA variables, identified in Table 2 in terms of their C-index performance. A first assessment consisted of statistical summaries of the sizes of the final predictive subsets selected by each pipeline, reported in Table 3. All clinical-only pipelines showed little deviation around a typical 5-variable model structure. In terms of the make-up of these models, however, the LASSO and Boosted Cox pipelines selected 14 clinical variables across all bootstraps, compared to 8 for traditional CPH and the RSF (Table 3), suggesting higher stability of the latter 2 for the clinical-only models. When including mRNA data, all models typically tended to contain around 10 to 16 variables for prediction. Remarkably, the typical final feature set size of Cox-based mRNA-inclusive pipelines had a coefficient of variation (CoV) either lower than, or comparable to that of clinical-only pipelines, indicating reasonably low variability in final model sizes for mRNA-inclusive models. An increase in CoV, however acceptable, was noted for RSF after introduction of mRNA information. This first analysis thus suggested that mRNA-inclusive pipelines tended to include a reasonable number of variables in their models.

**Figure 4:**
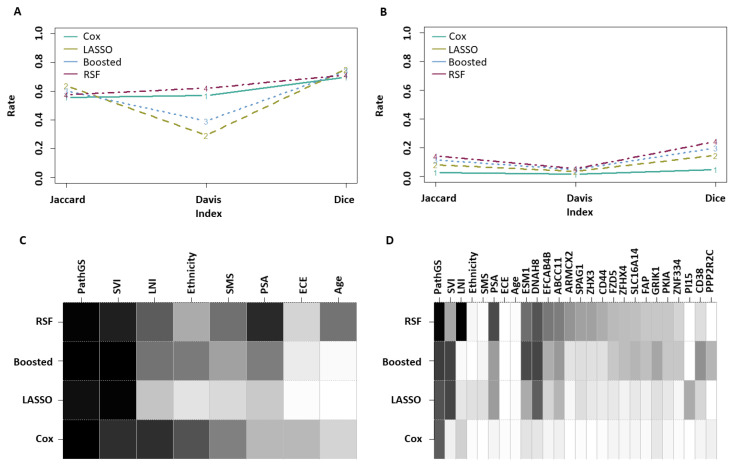
(**A**) Stability indices of the clinical-only models. (**B**) Stability indices of the top mRNA-inclusive pipelines. (**C**) Feature selection rates in clinical-only models (a darker shade indicates a higher rate). (**D**) Feature selection rates in the best-performing mRNA-inclusive pipelines, for features with a selection rate ≥0.2 in at least one of these top pipelines. For comparative purposes, the shade of grey used for ESM1 in RSF corresponds to a 50% selection rate.

Model stability was further measured by considering the overall individual feature selection rates across bootstraps, as well as on the basis of three overall stability indices [47], namely the Jaccard, Davis, and Dice indices, as shown in Figure 4. The Davis index in Figure 4A confirmed increased stability of the traditional Cox and RSF pipelines over the LASSO and Boosted Cox alternatives for clinical-only modelling. As for mRNA-inclusive pipelines, the comparison of metrics shown in Figure 4A with those of Figure 4B indicated lower stability, as would be expected given the sheer size of the genomic dataset. Finally, RSF outperformed the CPH pipeline when using mRNA, as shown in Figure 4B. In complement, the traces of overall feature selection rates shown in Figure 4C,D illustrate the tendency of RSF pipelines to yield higher model stability and feature selection rates overall compared to the LASSO and Boosted Cox models, whether using clinical-only or combined clinical and mRNA information.

In summary, this set of results clearly indicates that the RSF is a more stable pipeline than the LASSO and Boosted Cox alternatives. Whether considering clinical-only or clinical and mRNA features, the RSF yielded the most stable pipeline overall in the latter case. Moreover, the RSF and Boosted Cox methodologies, which were the most competitive overall, do not rely on a proportional hazard assumption. This may suggest that the Cox PH assumption is a possible limitation for the inclusion of mRNA information. Similarly, RSF does not constrain to linear interactions between the predictors, which may be a reason for its overall higher predictive performance. The overall lower performance seen with LASSO Cox, on the other end, may be due to the inability of this model to cope well with multi-collinearity or confounding, both of which are possibly present among the mRNA feature set. The differences in performance observed between the models must be confirmed in independent studies before any reliable insights can be derived on the specific aspects behind the performance of each model.

Overall, the pathological Gleason Score was the single-most prevalent predictor across all pipelines (Figure 4C,D; Table 4). It was the only variable that was selected more than 50% of the time in conventional Cox modelling. The other three modelling approaches had more stable individual feature selection rates. Figure 4C illustrates the consistent tendency by all clinical-only models to use pathological Gleason score and SVI in particular. This trend remained present in LASSO and Boosted Cox models following the introduction of mRNA data (Figure 4D and Table 4), whereas RSF tended to use LNI and PSA mainly alongside the Gleason score. Finally, two mRNA variables, namely DNAH8 and ESM1, appeared consistently in a number of pipelines. Both of these have been noted in other reports for their association with prostate cancer. DNAH8 has been shown to be a marker for poor prognosis in prostate cancer [49]. Elevated ESM1 has been shown to be associated with prostate cancer progression and metastasis [50].

## 4. Discussion

### 4.1. Key Findings

In this study, the machine learning pipelines developed for prediction of BCR-free survival using Cox proportional hazards and random survival forest models using clinical variables only outperformed the reference MSKCC nomogram with respect to model discrimination and calibration. This is in line with previous findings by Jeffers et al. where their RSF model with clinical variables has only been shown to improve on the Kattan nomogram [51]. Additionally, predictive performance of the models was enhanced by the addition of mRNA variables. The three best performing pipelines included both mRNA information and clinical variables, indicating the potential for genetic information to improve the predictive performance when combined with routine clinical variables. The mRNA features most frequently included in the models were DNAH8 (Dynein axonemal heavy chain 8) and ESM1 (Endothelial Cell Specific Molecule 1). The main function of DNAH8 is currently understood to be related to sperm motility [52] and has been shown to be a marker for poor prognosis in prostate cancer [49] and to be associated with metastasis [53]. Elevated ESM1 readings have been shown to be associated with prostate cancer progression and metastasis [50], ESM1 over-expression correlates to a higher Gleason Score [54]. Both of these variables were also found to be useful predictors in the pre-operative setting by this group in other analyses.

### 4.2. Other Markers of Interest

Four clinical variables were repeatedly retained in the models that included mRNA information; pathological Gleason sum, seminal vesicle invasion, preoperative PSA, and lymph node involvement. PSA was not consistently selected in the Cox-based models, but was in the RSF pipelines, which suggests that this antigen level may interact with predictors in a nonlinear way with respect to the endpoint of interest. None of the mRNA variables identified by Taylor et al. as tumour suppressors [25] were selected in our models. This may indicate a lack of association between tumour suppression and biochemical recurrence. Similarly, none of the variables used in the Decipher classifier for the prediction of metastasis following RP [22] were selected. These mRNA variables and their pathways, which are associated with metastasis, may be different to those involved in biochemical recurrence itself. Other non-genetic markers such as measures of systemic inflammation such as neutrophil-to-lymphocyte ratio (NLR) have also been investigated recently for their potential as predictive markers for BCR. Results on the prognostic potential of NLR for BCR prediction presented in the literature are mixed [55,56,57,58,59,60,61]. As the dataset used in this study did not contain this information, it was not possible to investigate the potential of NLR in conjunction with mRNA variables at this time, and it is therefore an avenue for future work.

### 4.3. Model Performance and Key Feature Identification

While the traditional CPH model with prefiltered mRNA variables demonstrated the best discrimination performance, its poorer calibration at specific endpoints as well as overall calibration suggests overfitting. On the other end, the best performing RSF-based pipeline outperformed other modelling strategies in terms of discrimination and calibration for 2-year, 5-year, and overall BCR-free survival prediction. The RSF model was also shown to have the highest AUC at both 2-year and 5-year horizons and gave rise to the most consistent net benefit at these time-points in the decision curve analysis. Among the most common methodologies available for survival analysis in continuous time, it may thus be a more opportune strategy overall compared to Cox-based approaches. Although the machine learning models were tested internally using a bootstrapping framework, these findings require external validation, which was not possible given the relatively small size of this dataset. Further improvement may also be possible using novel deep learning techniques, such as the Cox-based deep neural network recently described by Hao et al., 2021 [62], for application to high-dimensional data with small sample sizes. Experimentation of this recent innovation to BCR prediction for prostate cancer will be done in future work by this group.

Not all mRNA-inclusive pipelines yielded an increase in the predictive performance over their clinical-only counterpart. Whilst including mRNA variables in Boosted Cox models always led to a statistically significant increase in performance, regardless of feature pre-filtering and selection technique used, none of the LASSO Cox alternatives did. As for RSF pipelines, improvement required the combined use of correlation prefiltering and univariate feature selection. These findings are in line with those found by Gade et al. who investigated the inclusion of microRNA and mRNA data by using a bipartite graph as a single predictor [63]. The differences across the pipelines may be due to a large extent to the lack of statistical power associated with the relatively small sample size available, but it also indicates that the model building process is non trivial. Less than 2% of the features available were used at least once by a pipeline within the bootstrapping framework, and a gain in both model stability and predictive performance may be achieved with feature pre-processing strategies that are better suited to the nature of these data.

Moreover, unknown potential predictors of BCR may have been inadvertently removed from the pool of candidates prior to model building, e.g., due to the inability of feature selection methods based on multiple univariate selection to identify variables that may provide additional predictive potential only when used in conjunction with other variables. Other methods of pre-filtering and feature elimination will be investigated in future work including the potential benefit of clustering and other relevant methods such as principal component analysis.

## 5. Conclusions

The ability to predict BCR-free survival is critical for therapeutic decision-making. After comparing several machine learning methodologies with the reference Memorial Sloan Kettering nomogram, the main finding of this study was that the inclusion of mRNA variables increased the predictive performance. The best-performing pipeline combined correlation based pre-filtering and univariate feature selection of mRNA variables with a random survival forest model. It yielded high discriminative performance while retaining satisfactory calibration at 2-year, 5-year and overall survival, as well as the highest AUC and net benefit across all pipelines benchmarked. mRNA variables ESM1 and DNAH8 were consistently found to be strong predictors for 2-year, 5-year, and overall BCR-free survival.

## Figures and Tables

**Table 1:** Descriptive statistics of the categorical clinical variables.

Variable	N	%
**BCR-free Survival**		
Censored	135	72
BCR	52	28
**Ethnicity**		
White	147	79
Black	30	16
Asian	4	2
Unknown	6	3
**Pathological Gleason Score**		
6	52	28
7	103	55
8	17	9
9	15	8
**Extracapsular Extension (ECE)**		
Absent	52	28
Present	135	72
**Seminal Vesicle Invasion (SVI)**		
Negative	156	83
Positive	31	17
**Lymph Node Involvement (LNI)**		
Normal	140	75
Abnormal	19	10
Not Done	28	15
**Surgical Marginal Status (SMS)**		
Negative	137	73
Positive	50	27

**Table 2:** Bootstrapped C-indices. The top performing pipelines for each statistical model (namely CPH, LASSO Cox, Boosted Cox, and RSF) are highlighted in bold.

Model	C-Index	95% Confidence Interval
Memorial Sloan Kettering (MSKCC)	0.770	(0.675, 0.844)
Clinical Variables Only		
Cox	0.798	(0.724, 0.857)
LASSO	0.761	(0.710, 0.824)
Boosted	0.770	(0.709, 0.829)
RSF	0.795	(0.727, 0.852)
Clinical Variable and Correlation Prefiltered mRNA variables		
**Cox **	**0.828 **	**(0.720, 0.909) **
LASSO	0.762	(0.696, 0.826)
**Boosted**	**0.816**	**(0.757, 0.866)**
RSF	0.749	(0.695, 0.811)
Clinical Variables and Univariate Cox Feature Selected mRNA variables		
Cox	0.765	(0.688, 0.827)
**LASSO**	**0.768**	**(0.703, 0.835)**
Boosted	0.798	(0.737, 0.845)
RSF	0.782	(0.730, 0.846)
Clinical Variables and Correlation Prefiltered and Univariate Cox Feature Selected mRNA variables		
Cox	0.792	(0.671, 0.865)
LASSO	0.763	(0.464, 0.918)
Boosted	0.814	(0.753, 0.863)
**RSF**	**0.821**	**(0.747, 0.874)**

**Table 3:** Summaries of final predictive model sizes (i.e., number of variables used) selected within the clinical-only models and best-performing pipelines, in terms of their mean, median, standard deviation, and coefficient of variation (CoV), as well as the overall number of features selected at least once across 100 bootstraps.

	Mean	Median	SD	CoV	Overall
**Clinical-only**					
Cox	4.55	5	1.250	27.5%	8
LASSO	3.51	3	1.541	43.9%	14
Boosted	5.10	5	0.870	17.1%	14
RSF	4.96	5	1.524	30.7%	8
**Top performing pipelines**					
Cox	9.46	10	0.926	9.8%	623
LASSO	11.88	12	3.891	32.8%	343
Boosted	14.98	15	3.052	20.4%	323
RSF	17.85	16	9.057	50.7 %	335

**Table 4:** Selection rates of all variables with a selection rate ≥ 50% in at least one of the best-performing mRNA-inclusive pipelines.

	Pathological Gleason Sum Score	LNI	SVI	PSA	DNAH8	ESM1
Cox	56%	16%	5%	4%	9%	10%
LASSO	60%	9%	64%	34%	55%	29%
Boosted	67%	2%	65%	34%	64%	62%
RSF	86%	88%	31%	62%	59%	50%

## Data Availability

Publicly available datasets were analysed in this study (accessed 14 June 2021). This data can be found here: https://www.ncbi.nlm.nih.gov/geo/query/acc.cgi?acc=GSE21032.

## Abbreviations

The following abbreviations are used in this manuscript:

BCR	Biochemical recurrence
mRNA	Messenger ribonucleic acid
RP	Radical prostatectomy
PSA	Prostate specific antigen
CPH	Cox proportional hazard
ECE	Extracapsular Extension
SVI	Seminal vesicle invasion
LNI	Lymph node involvement
MSKCC	Memorial Sloan Kettering Cancer Centre
